# Health systems analysis of eye care services in Zambia: evaluating progress towards VISION 2020 goals

**DOI:** 10.1186/1472-6963-14-94

**Published:** 2014-02-28

**Authors:** Fiammetta Maria Bozzani, Ulla Kou Griffiths, Karl Blanchet, Elena Schmidt

**Affiliations:** 1 Department of Global Health and Development, London School of Hygiene and Tropical Medicine, 15-17 Tavistock Place, WC1H 9SH London, UK; 2 International Centre for Eye Health, London School of Hygiene and Tropical Medicine, London, UK; 3 Sightsavers, Haywards Heath, West Sussex, UK

**Keywords:** Eye care, Health systems analysis, VISION 2020

## Abstract

**Background:**

VISION 2020 is a global initiative launched in 1999 to eliminate avoidable blindness by 2020. The objective of this study was to undertake a situation analysis of the Zambian eye health system and assess VISION 2020 process indicators on human resources, equipment and infrastructure.

**Methods:**

All eye health care providers were surveyed to determine location, financing sources, human resources and equipment. Key informants were interviewed regarding levels of service provision, management and leadership in the sector. Policy papers were reviewed. A health system dynamics framework was used to analyse findings.

**Results:**

During 2011, 74 facilities provided eye care in Zambia; 39% were public, 37% private for-profit and 24% owned by Non-Governmental Organizations. Private facilities were solely located in major cities. A total of 191 people worked in eye care; 18 of these were ophthalmologists and eight cataract surgeons, equivalent to 0.34 and 0.15 per 250,000 population, respectively. VISION 2020 targets for inpatient beds and surgical theatres were met in six out of nine provinces, but human resources and spectacles manufacturing workshops were below target in every province. Inequalities in service provision between urban and rural areas were substantial.

**Conclusion:**

Shortage and maldistribution of human resources, lack of routine monitoring and inadequate financing mechanisms are the root causes of underperformance in the Zambian eye health system, which hinder the ability to achieve the VISION 2020 goals. We recommend that all VISION 2020 process indicators are evaluated simultaneously as these are not individually useful for monitoring progress.

## Background

In 2010 approximately 39 million people worldwide were blind and 285 million were visually impaired [1]. Around 90% of the burden was in low-and middle-income countries and it was estimated that 75% of cases could be prevented or cured [1]. “VISION 2020: The Right to Sight” is an international initiative launched in 1999 by the World Health Organisation (WHO) and the International Agency for the Prevention of Blindness (IAPB). The aims are to eliminate avoidable blindness by 2020 and prevent the projected doubling of the burden of visual impairment between 1990 and 2020 [2]. Focus is placed on targeting the leading causes of blindness: cataract, trachoma, onchocerciasis, childhood blindness and refractive error [3].

Comprehensive eye care services include eye health promotion, prevention, treatment and rehabilitation. An essential prerequisite for achieving the VISION 2020 goals is that these services are well integrated into national health systems. To date, all 193 WHO member states have formally pledged to invest in eye care and the large majority of countries have formed VISION 2020 committees and drafted national eye care plans [4]. However, the implementation of these plans varies widely across countries and remains the biggest challenge for reaching the set goals.

The Zambian government signed the VISION 2020 global declaration in 2004 and launched a national eye care programme the same year. The objectives of the present study were to undertake a situation analysis of Zambian eye health services and examine progress towards achieving VISION 2020 process indicator targets. The analysis should be used by Zambian policy makers to facilitate future planning of eye care services and by international stakeholders when evaluating progress and constraints for achieving VISION 2020 goals. In addition, the present study seeks to contribute to methodological issues in health systems analysis.

## Methods

### Data collection

Data were collected using a mixed methods approach between February and June 2011 from three sources: (i) A questionnaire-based survey of representatives of all eye care facilities in Zambia, (ii) semi-structured interviews with five key informants working in eye care, and (iii) review of national strategic plans and policy documents related to health and eye care in Zambia. Triangulation was used to synthesise and integrate the data from multiple sources [5,6].

The survey questionnaire was adapted from the VISION 2020 situation analysis data collection tool and from a list of essential equipment for a functional eye unit compiled by IAPB [7,8]. Data collected included sources of funding, ownership, types of services delivered, human resources, equipment and number of beds available for ophthalmic patients. To identify survey participants, directories of public and private facilities offering eye care services were obtained from the Ministry of Health, The Church Health Association of Zambia and the Health Institutions and Professionals Board. These lists were verified and supplemented with information from key informants to ensure that no providers were missed. The questionnaire was emailed to facilities for self-completion after a preliminary telephone call or administered during face-to-face interviews. A total of 74 respondents, one from each eye care facility identified, took part in the survey.

Five key informants were purposively selected on the basis of their knowledge of the Zambian eye care sector and interviewed face-to-face following a semi-structured topic guide. Questions concerned current levels of service delivery, the relationships between different actors in the sector, and opportunities and constraints. The key informants were all from the main urban areas (Lusaka and Copperbelt), occupying positions within the Ministry of Health, the main public and private eye care facilities and the University of Zambia. Handwritten notes were taken during the interviews, which lasted on average 20 minutes.

### Analytical frameworks

VISION 2020 process indicators were calculated for Zambia and used for assessing the status of human resources and service delivery (Table 1). These indicators were established by a WHO expert group in 1997 and based on the best epidemiological evidence available at the time, combined with a pragmatic approach as to what were achievable targets for 2010 and 2020 [8,9]. Higher target levels were established for Asia than Sub-Saharan Africa because of the higher population density and percentage of population above sixty years of age in this region. While some efforts have been made by the WHO to monitor the VISION 2020 process indicators, this has not been done systematically and, to our knowledge, the present study is the first to evaluate the indicators in depth for a specific country.

**Table 1:** VISION 2020 process indicators for Sub-Saharan Africa

**Human resources:**	
**●**	One ophthalmologist per 250,000 population
**●**	One ophthalmic clinical officer (OCO) per 200,000 population
**●**	One ophthalmic nurse (ON) per 200,000 population
**●**	25% of secondary eye facilities should employ a full-time manager
**●**	25% of secondary eye facilities should employ an equipment technician
**Services:**	
**●**	One eye bed per 20,000 population
**●**	One eye operating theatre per million population
**●**	One spectacles manufacturing workshop per million population
**Outputs:**	
**●**	4,000 cataract surgeries per year per million population

Source: WHO 2002 [8].

**Figure 1:**
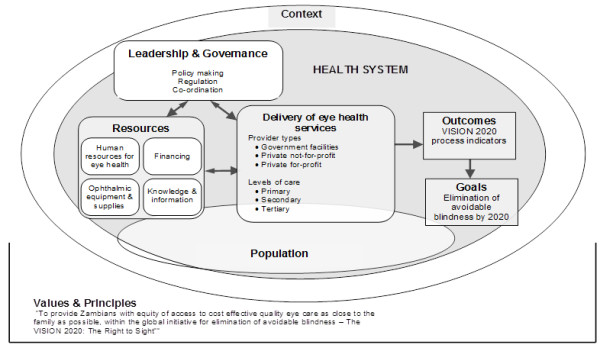
**Health system dynamics analysis framework adapted to eye health care in Zambia.** Adapted from van Olmen *et al.*, [10].

The health system dynamics framework developed by van Olmen *et al*. was adapted to analyse eye health services in Zambia (Figure 1) [10]. The framework identifies ten health system components: 1) values & principles, 2) goals & outcomes, 3) the context, 4) leadership & governance, 5) service delivery, 6-9) organisation of resources (human resources, financing, infrastructure & supplies, knowledge & information), and 10) the population. Some of these elements also appear in other frameworks, such as the WHO health system building blocks, which have been adapted to eye health [11,12]. A novelty introduced by van Olmen *et al.* is the focus on linkages between different elements of the system, emphasising how outcomes and goals are achieved as a result of complex interactions between all ten components. The health system dynamics framework was used to map and chart information from different sources in a thematic content analysis, whereby data are presented following the components of the framework. Success or failure in different components was gauged by evaluating relevant VISION 2020 process indicators wherever possible.

Ethics approval was obtained from the London School of Hygiene and Tropical Medicine and the University of Zambia Research Ethics Committee.

## Results

We found substantial shortcomings in all components of the health system dynamics framework and weak linkages between the elements. Our survey identified major deficiencies in VISION 2020 process indicators, but routine data on outputs and outcomes were extremely sparse. Below we present the findings according to each health system component, followed by an analysis of linkages between elements.

### Values and principles: as stipulated by government health sector plans

The first Zambian eye health strategic plan covered the period 2006-2011 and the second is for 2012-2015 [13,14]. Both plans share a vision to provide Zambians with “equity of access to cost effective quality eye care as close to the family as possible, within the global initiative for elimination of avoidable blindness–The VISION 2020: The Right to Sight”. The overall intent of the Zambian health system is to promote “a society in which Zambians create environments conducive to health, learn the art of being well, and provided with basic health care for all” [15].

### Goals and outcomes: as specified in the eye health strategic plan

The eye health strategic plan for 2012-2015 outlines five goals; (i) raising awareness of avoidable blindness in primary care, (ii) provision of services against diseases that cause blindness, (iii) provision of eye surgery, (iv) production of low cost spectacles and (v) incorporation of community-based rehabilitation in the provision of comprehensive eye services [13].

### Context: human resources crises

Despite reporting some health gains since the 1990s, health outcomes remain poor in Zambia and the health-related Millennium Development Goals are unlikely to be achieved by 2015 [16]. The largest obstacle is a human resources crisis that has grown out of proportions, even compared to other low-income countries. A recent study on the Zambian human resources for health found that the country has one health worker for every 1,000 people, which is less than Benin (1.1), Rwanda (1.2), Ghana (1.9), and India (1.9), and is well below the internationally recommended benchmark of 2.3 health workers per 1,000 population [17,18]. Moreover, just a little over half of all health workers are clinical cadres and, of these, more than 60% are nurses and only 7% are doctors. In 2008, the number of doctors in Zambia was just 42% of the national benchmark, the number of nurses 46%, and the number of midwives 48% [17]. The key reasons for the inadequate number of health workers were low levels of new trainees, premature death of staff due to HIV/AIDS, and emigration, particularly among doctors. The fraction of Zambia-trained doctors working abroad was close to 60%, compared to an average of 28% for all Sub-Saharan African countries combined [19].

### Leadership & governance: administrative structures

Within the Ministry of Health, the Office of the National Eye Care Coordinator was created in 2006. Its principal role is to coordinate international partners working in eye health, with the aim of avoiding duplication and ensuring that operations conform to the priorities laid out in the strategic plan. This oversight, however, does not extend to the private for-profit sector, and in the public sector, coordination and enforcement of regulations are decentralised to the districts, but no specific budget for eye care is in place at this level.

The Ministry of Community Development, Mother and Child Health is responsible for formulating disability-related policies and the Zambia Agency for Persons with Disabilities coordinates their implementation and acts as an advisory body to the ministries [20]. Two disabled people’s organisations represent visually impaired people.

### Delivery of eye health services: survey findings

A total of 74 facilities offered eye care services in 2011. Of these, 43% were government owned, 22% were owned by Non-Governmental Organizations (NGOs) and 35% were private for-profit facilities (Table 2). Nineteen of the 32 government facilities received support from international NGOs, mainly Sightsavers, Vision Aid Overseas, Operation Eyesight and the Christian Blind Mission. The majority of private for-profit providers (73%) were optical shops.

**Table 2:** Ownership structure and level of care of eye care facilities

		**Level**		
**Ownership**	***Primary***	***Secondary***	***Tertiary***	**Total**
Government (with NGO support)	2 (2)	28 (15)	2 (2)	32 (19)
NGO	4	11	1	16
Private for-profit	19	6	1	26
**TOTAL**	25	45	4	74

*Primary:* Diagnostic, refractive and referral services. Optical shops in the private sector.

*Secondary:* Basic surgical procedures, including cataract, trichiasis and glaucoma.

*Tertiary:* Advanced surgical procedures, including vitreo-retinal surgery and laser photo-coagulation.

**Figure 2:**
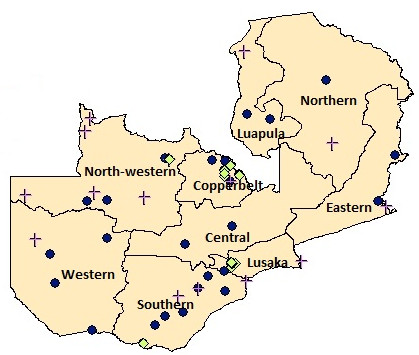
**Map of Zambia.** Legend: yellow green diamond, Private for-profit facility; purple cross, Mission facility; dark blue circle, Government facility.

The distribution of eye care facilities according to ownership is shown in the map in Figure 2. Half of all facilities were located in Lusaka (28%) and in the Copperbelt (22%), two highly urbanised provinces, which jointly account for 28% of the population (Table 3). In contrast, in the rural Northern Province, 3% of all eye care facilities served 13% of Zambia’s population. While government facilities were evenly distributed across the country, the private sector operated exclusively in the urban areas of Lusaka, the Copperbelt, North-Western and Southern provinces. Conversely, NGOs were predominantly located in remote districts, such as Nchelenge in Luapula or Kalabo in Western province, where they represented the only source of eye health services for vast catchment areas.

**Table 3:** VISION 2020 process indicators according to province

**Province**	Share of total pop.* (%)	Monthly per capita income (2010 US $)*	**Number of eye health facilities**	**Eye surgery theatres per million pop.**	**Spectacles workshops per million pop.**	**Eye beds per 20,000 pop.**	**Ophthalmologists per 250,000 pop.**	**OCOs per 200,000 pop.**	**ONs per 200,000 pop.**	**% facilities with eye unit manager**	**% facilities with technician**
Lusaka	13	95	21	2.73	0.91	0.79	0.45	0.64	1.09	19.1	19.0
Copperbelt	15	92	16	2.55	0.05	2.45	0.64	0.82	1.02	6.2	6.2
Northern	13	33	2	0.57	0.00	2.73	0.14	0.11	0.00	0.0	0.0
Eastern	14	30	4	1.76	0.00	5.95	0.15	0.35	0.12	0.0	0.0
Southern	13	57	11	1.24	0.62	8.86	0.47	0.87	0.50	36.4	0.0
Central	11	45	1	0.79	0.00	0.03	0.20	0.16	0.16	0.0	100.0
Luapula	8	28	3	2.09	0.00	0.63	0.26	0.42	0.00	66.7	33.3
Western	7	35	6	2.27	0.00	1.47	0.28	0.91	0.45	0.0	0.0
North-Western	6	48	10	2.83	1.42	1.61	0.35	0.57	0.85	0.0	0.0
**Zambia**	100	55	74	2.15	0.38	2.98	0.34	0.54	0.51	14.9	10.8

*Source: Living conditions monitoring survey 2006 & 2010 [21].

OCO: Ophthalmic clinical officer.

ON: Ophthalmic nurse.

VISION 2020 target for process indicators = 1 for ratios and 25% for proportions.

A total of 1,941 beds were available for ophthalmic patients. Of these, 217 were for eye patients only, while the remainder were in general wards and used on a needs basis. This translated to a national figure of approximately three beds per 20,000 population, exceeding the VISION 2020 goal of one bed for every 20,000 people. However, as shown in Table 3, there was great regional variation, with Central and Luapula provinces remaining below target.

Although 70% of facilities in rural areas reported offering refraction and essential surgeries, such as cataract and trichiasis, in 68% of facilities offering these services the procedures were only performed a few times a year by visiting outreach teams from larger, urban facilities, bringing their own equipment. Complex services, such as vitreo-retinal surgery and laser photo-coagulation, were only available at four facilities in the wealthier parts of the country, namely at one government facility in the Copperbelt and at one NGO, one government and one private for-profit hospital in Lusaka. International NGOs provided financial and technical assistance to these two government tertiary facilities.

Only seven corrective spectacles manufacturing workshops were in operation: two of them were in Lusaka, one in the Copperbelt and four in the main towns of North-western, Central and Luapula provinces. All the workshops were established by international NGOs (three of them located at NGO facilities and the remaining four within government facilities) and operated commercially with the ultimate aim of self-sustainability. The monthly output of made-to-order (no stock policy) prescription glasses ranged from 100 pairs of spectacles in the Lusaka and Copperbelt workshops to around 50 pairs in the rural provinces.

Data for calculating the cataract surgical rate are not routinely collected and reported. In the 2006-2011 strategic plan, the rate was estimated at 750 per year per million population, with a target of 1,500 for 2011 [14]. In the second strategic plan for 2012-2015, the 2011 rate was estimated at 1,500 and the target for 2015 was 2,000 per year per million population [13]. However, no data confirming the achievement of the 2011 target or any provincial variations in the rate were presented. A Rapid Assessment of Avoidable Blindness (RAAB) survey in Southern Province estimated the cataract surgical coverage to be 46% of the population in need above 50 years of age [22]. In this survey, 3,629 people above 50 years of age were randomly selected to have their eyes tested. The prevalence of blindness was 2.3% (Visual Acuity (VA) < 3/60 in the better eye with available correction) and 8.7% were visually impaired (3/60 ≤ VA ≤ 6/18 in the better eye with available correction). The conditions with the highest prevalence among the study population were untreated cataract (47%), uncorrected refractive error (20%), posterior segment disease (19%) and corneal scarring (10%).

### Resources

#### Human resources for eye health: survey findings

A total of 191 people worked full time in Zambian eye care in 2011. The VISION 2020 ratios to population for ophthalmologists and ophthalmic clinical officers (OCOs) were higher in urban, high-income areas than in rural areas, but below the recommended target in every province (Table 3). Of the 18 ophthalmologists actively engaged in clinical practice at the time of the survey, four were based in Lusaka, five in the Copperbelt and three in Southern Province. These three provinces, representing 41% of the total population, retained 66% of ophthalmologists, 62% of cataract surgeons, 63% of OCOs, 75% of refractionists, 79% of ophthalmic nurses (ONs) and 95% of optometrists. The proportion of secondary facilities with a full-time manager was 19% in Lusaka, 9% in Southern province, 6% in the Copperbelt and zero in all other provinces. Maintenance technicians operated in 11% of facilities.

As shown in Table 4, 53% of all human resources were based at Government facilities. All optometrists but one worked at private-for-profit optical shops. Eighty-six percent of clinical cadres (ophthalmologists, OCOs, ONs and general nurses) were based at facilities fully or partially supported by NGOs and 11% of staff in these roles also worked as consultants in private practice.

**Table 4:** Socio-demographic characteristics of human resources for eye health in Zambia, 2011

**Position**	**No.**	**Government facilities**	**NGO facilities**	**Private for-profit facilities**	**Female (%)**	**Zambian (%)**	**Years of practice, mean (SD)**
Ophthalmologist	18	12	3	3	28%	72%	9.3 (7.7)
Ophthalmic clinical officer	35	23	8	4	23%	97%	7.2 (9.2)
Ophthalmic nurse	33	26	4	3	63%	97%	8.1 (8.8)
General nurse	47	25	20	2	49%	100%	5.5 (4.2)
Nursing aide	8	0	7	1	25%	100%	7.3 (5.7)
Cataract surgeon	8	4	1	3	0%	57%	9.1 (9.2)
Optometrist	19	1	0	18	0%	11%	10.6 (7.1)
Refractionist	4	2	0	2	50%	100%	4.3 (5.3)
Manager	11	5	4	2	55%	91%	5.6 (4.3)
Maintenance technician	8	4	1	3	0%	88%	7.6 (10.4)
**TOTAL**	191	102	48	41	36%	86%	8.8 (8.7)

Until recently, there were no training courses for ophthalmologists, OCOs and ONs available in Zambia. For this reason, although 79% of eye care professionals were Zambians, as many as 39% had completed their training abroad, including in Germany, the United Kingdom, Kenya, Malawi, Tanzania and the Gambia. This situation is gradually changing with the creation of diplomas for OCOs, ONs and optometrists at Chainama College of Health Sciences in Lusaka and a specialisation in ophthalmology at the University of Zambia Medical School available from 2011.

#### Equipment and supplies: survey findings

Thirty-nine percent of all facilities reported availability of functional instruments required for cataract diagnosis and follow-up (slit lamp, ophthalmoscope and retinoscope). For the remaining 61%, this equipment was only available when outreach teams visited from larger facilities or from overseas. While 80% of all secondary and tertiary eye units had an operating microscope, only 42% had a functioning A-scan for biometry, thus increasing the likelihood that patients might need refraction post-surgery. Six percent of all facilities had no functioning tonometers to measure intra-ocular pressure in glaucoma patients. Lusaka, the Copperbelt and Southern Province combined commanded 72% of the basic eye care equipment.

Ten percent of all instruments were either in need of or beyond repair. The items most frequently reported to be in less than optimal conditions were direct ophthalmoscopes (19%), slit lamps (18%), cataract sets (15%), and trial lenses and frames (11%). The most common reasons were lack of maintenance technicians and, especially in the case of donated equipment, absence of trained personnel able to set up and operate the instruments. The proportion of out-of-order instruments was 50% in government eye units, 47% in NGO and 3% in for-profit facilities.

#### Financing: survey findings

In 2006, user fees were abolished in all public primary care facilities located in rural areas, i.e. in 57 out of 72 districts [23]. The ultimate aim is to abolish user fees in all primary care facilities [24]. As shown in Table 2, there were only two primary care government facilities delivering eye services in rural areas, while the rest were either higher level facilities (40%) or located in urban areas (59%). Moreover, 14 out of 30 rural facilities were owned by NGOs and only 3 of them waived user fees for some services. Hence, only approximately 25% of all eye care facilities in Zambia provided services for free at the point of use. A study has shown that after user fees were removed in rural primary facilities, health service utilisation levels among the rural population increased and exceeded the rate for the same group in the urban population [25].

The “low cost” fee for cataract surgery in one eye was approximately US$ 100 in private and NGO hospitals and US$ 20 at government facilities. “High-cost” patients, receiving surgery with less waiting time and in a more comfortable environment, paid approximately US$ 500 in private practice and US$ 100 at government facilities, respectively. A pair of prescription glasses from an NGO manufacturing workshop cost between US$ 2 and US$ 165, while prices at private optical shops ranged between US$ 100 and US$ 400.

#### Knowledge & information: document review

No routinely published data sources on eye disease prevalence, service usage and outputs were available. The Health Management Information System of the Ministry of Health includes procedures for collecting data on glaucoma, refractive error, allergic conjunctivitis and other eye infections presenting at primary care facilities from OPD tally sheets [26]. However, these data are not routinely analysed or published and other relevant eye conditions, most notably cataract, are missing from the manual altogether. The most recent versions of the National Health Strategic Plan, the Demographic and Health Survey and WHO country reports did not provide information on eye conditions [15,27,28].

### The population: document review

In eye care, an example of population involvement in supply of services are volunteer community eye workers (CEWs), who can act as focal points for information on available ophthalmic services close to the community and can actively assist with case finding, diagnosis and drugs distribution, especially for trachoma initiatives [29,30]. In Zambia, approximately 150 CEWs have been trained by NGOs in case finding and referral, but no official records of their activities were available.

Service usage is a function of both supply and demand [10]. On the supply side, we found access to eye care services constrained by the physical lack of providers in remote parts of the country and by the financial barriers created by the user fees charged in 75% of all facilities [25]. However, according to RAAB survey findings in Southern Province, demand-side reasons for not accessing cataract surgery were not just financial, but included lack of awareness of treatment availability (36%), a belief that blindness was God’s will (16%), no information on how to get surgery (12%) and no services locally available (11%) [22].

### Linkages between health system elements

Several gaps were evident in all areas of the health system dynamics framework. Most importantly, *resources* were scarce and inadequately distributed. There was a shortage of skilled human resources, particularly ophthalmologists and cataract surgeons in operating theatres. Equipment was mostly concentrated in urban areas, often in a state of disrepair and personnel who could operate and maintain it was lacking. The financing model was not sustainable and inequitable, as it largely relied on out-of-pocket payments from a population who can hardly afford to pay. A case in point, linking resource scarcity to inadequate financing mechanisms, was that of glasses, which had to be paid for directly by patients in the majority of cases and the number of manufacturing workshops producing affordable spectacles was too low to meet demand. Data on the prevalence of eye conditions and eye health system performance were lacking. This led, in turn, to difficulties with *leadership and governance* in terms of evidence based decision-making. All of these factors contributed to hampering *service delivery,* which ultimately made the eye health system unable to fulfil its *values and principles* and achieve the stated *goals and outcomes*.

## Discussion

Our study has showed several gaps in progress towards achieving the VISION 2020 goals in Zambia. Most importantly, the ratio of human resources for eye health to population is considerably below target for all clinical cadres and the percentage of eye care facilities employing managers and maintenance technicians is below the 25% goal. In addition to the scarcity of eye care staff, human resources are also unequally distributed across the country, favouring wealthier urban areas. Shortages and mal distribution of eye care staff, which are particularly severe for high-level clinical cadres, are in line with reports on the ongoing human resources crisis affecting the Zambian health system: surveys conducted in 2006 found that only 23% of all Zambian doctors worked in rural areas against 60% of clinical officers, and rural vacancies were more difficult to fill and were characterised by high levels of staff turnover [17,31]. Ferrinho and colleagues concluded that training more staff is necessary to address the crisis, but it is not sufficient and has to be complemented with measures to mitigate attrition and increase productivity [32]. The recently established training courses for high-and mid-level eye care cadres have greatly improved the situation in recent years and the VISION 2020 target for ONs is now met in two provinces. The shortage of human resources was accompanied by a scarcity of equipment and supplies that, once again, was most severe in remote districts.

The barriers encountered in accessing eye care services in rural areas were highlighted by all VISION 2020 indicators, particularly spectacles manufacturing workshops and eye surgery theatres per unit of population. Higher values for the indicators in urban areas were generally due to a larger presence of the private sector. Barriers to access were compounded by the current structure of health care financing in Zambia. The presence of user fees at the facilities where most eye health services were delivered added to other expenses such as transport and lost wages, which can be particularly high for rural residents who have to travel long distances to seek treatment [25]. Supply of prescription glasses is an example of a basic service that is relatively costly and often inaccessible outside the largest towns. For meeting VISION 2020 targets, it is imperative that the number of manufacturing workshops, where a pair of spectacles costs on average ten times less than at a private optical shop, be increased. During our fieldwork we found that donations of recycled prescription glasses from international and church-based NGOs were common in rural areas. However, research in other settings has demonstrated that donated spectacles are frequently in less than optimal conditions and, even when they are physically intact, the chance of finding the right prescription for each patient is small and uptake is limited because they are cosmetically inappropriate [33]. For these reasons, the Zambia Ministry of Health opposes the practice and other sustainable alternatives for supplying glasses to underserved areas are urgently needed.

The VISION 2020 process indicators were established in 2002 but, to our knowledge, they have not been monitored closely in any country. We found that the VISION 2020 service delivery indicators, such as the number of eye beds and eye surgery theatres per unit of population, were of limited value when viewed individually. Achieving the target number of eye beds is only useful if skilled human resources are also available; the beds will be empty if there is no staff to perform surgeries. Similarly, in areas where eye surgeries are only performed by visiting ophthalmologists, the target of one eye operating theatre per million population may present a more positive picture than the reality of day-to-day service delivery. Human resource indicators can also overestimate capacity if absenteeism and the practice of working at multiple facilities to earn extra income, both documented in Zambia, are not taken into account [17,31]. Hence, relying on just a selection of VISION 2020 process indicators to monitor progress may be misleading. We believe that the present study can contribute to a revitalisation and re-assessment of the process indicators and we urge the international eye health community to become more convincingly engaged in systematic monitoring of progress towards the VISION 2020 goals.

In a review by Ackland published in 2012, it was argued that the VISION 2020 initiative has raised awareness of eye health issues, increased the level of funding from corporate and Government sectors, and enabled formulation of national eye health strategic plans [34]. However, to reach the goals the remaining eight years need to focus on increasing advocacy at the national level and integrating eye care into health systems [34]. In this study we have offered a country-specific and system-wide perspective on VISION 2020.

Our study had a number of limitations. Firstly, our survey could have been subject to bias due to selective under-reporting as, due to practical constraints, some of our respondents were emailed the survey for self-completion while others responded face-to-face. We tried to minimise bias by contacting all facilities via telephone prior to emailing the questionnaire, to ensure that the same information was provided to all respondents and potential questions were answered. Secondly, we found it challenging to analyse our findings in an interlinked and dynamic manner according to the framework. This is a problem encountered by other health systems researchers [35]. While numerous frameworks have been developed to depict the structure of health systems (Hoffman and colleagues identified 41 health systems frameworks developed since 1972 [36]), major advances are still needed on how to actually use these frameworks in applied research. We have not been able to find other examples of studies in which our chosen health systems dynamics framework has been applied and we interpret this as an indication of the methodological hurdles still being faced in this area of research.

## Conclusions

We found that the Zambian eye health system is underperforming in all key areas of the dynamic analytical framework: lack of physical, financial and informational resources undermines leadership and governance and negatively affects delivery of eye health services to the population. The system’s inability to implement its values and principles of providing Zambians with equity of access to eye care to eliminate avoidable blindness translates into high prevalence of eye conditions and poor performance on the VISION 2020 process indicators. In particular, the human resources crisis and the skewed distribution of resources favouring urban areas affects performance on the indicators in the rural areas, where the majority of the Zambian population resides.

## Abbreviations

AIDS: Acquired immunodeficiency syndrome; CEW: Community eye worker; HIV: Human immunodeficiency virus; IAPB: International agency for the prevention of blindness; NGO: Non-Governmental Organisation; OCO: Ophthalmic clinical officer; ON: Ophthalmic nurse; OPD: Out-patient department; RAAB: Rapid assessment of avoidable blindness; VA: Visual acuity; WHO: World Health Organisation.

## Competing interests

The authors declare that they have no competing interests.

## Authors’ contributions

FB carried out data collection and analysis and drafted the manuscript. UG provided the original concept for the study, participated in its design and coordination and helped to draft the manuscript. ES and KB contributed to study design and to subsequent versions of the manuscript. All authors read and approved the final submission.

## Pre-publication history

The pre-publication history for this paper can be accessed here:

http://www.biomedcentral.com/1472-6963/14/94/prepub
